# Anti-Tumor Efficacy of a Mesothelin-Based Nanovaccine in a KPC Orthotopic Mouse Model of Pancreatic Cancer

**DOI:** 10.3390/vaccines13030314

**Published:** 2025-03-14

**Authors:** Daniele P. Ferrari, Özmen Çobanoglu, Sana Sayedipour, Omar Luna, Sonia A. M. Ferkel, David Agorku, Yomkippur Perez, Luis J. Cruz, Fernando Albericio, François Trottein, Frauke Alves, Marietta Andrea Markus, Fernanda Ramos-Gomes

**Affiliations:** 1 Translational Molecular Imaging, Max-Planck-Institute for Multidisciplinary Sciences, 37075 Göttingen, Germany; 2 Univ. Lille, CNRS, INSERM, CHU Lille, Institut Pasteur de Lille, U1019-UMR 9017-CIIL-Center for Infection and Immunity of Lille, F-59000 Lille, France; 3 Department of Radiology, Leiden University Medical Center, 2333 ZA Leiden, The Netherlands; 4 Department of Organic Chemistry, University of Barcelona, 08028 Barcelona, Spainalbericio@ub.edu (F.A.); 5 Miltenyi Biotec B.V. & Co. KG, 51429 Bergisch Gladbach, Germany; 6 Polypure AS, 1364 Fornebu, Norway; yomkippur@polypure.no; 7 Department of Haematology and Medical Oncology, Translational Molecular Imaging, University Medical Center Göttingen, 37075 Göttingen, Germany; 8 Institute of Diagnostic and Interventional Radiology, Translational Molecular Imaging, University Medical Center Göttingen, 37075 Göttingen, Germany

**Keywords:** pancreatic cancer, immunotherapy, CD8^+^ T cells, mesothelin, nanoparticles

## Abstract

**Background/Objectives**: Immunotherapy has shown promising results in some cancers, but its efficacy remains limited in pancreatic ductal adenocarcinoma (PDAC). Vaccines in nanoparticle form (nanovaccines) can incorporate immunostimulating components to induce a potent immune response. As mesothelin (MSLN) is a tumor-associated antigen overexpressed in PDAC, we evaluated the effect of MSLN nanovaccine in a syngeneic orthotopic KPC-PDAC mouse model. **Methods**: An MSLN peptide combining three MSLN epitopes and two adjuvants, poly I:C and R848, was encapsulated in PLGA–chitosan nanoparticles to generate the nanovaccine. **Results**: The MSLN nanovaccine was successfully taken up by dendritic cells in vitro and was found in inguinal lymph nodes 24 h after subcutaneous injection into C57BL/6 mice. Nanovaccine re-stimulation of splenocytes from vaccinated mice led to increased levels of interferon-γ in vitro compared to unstimulated splenocytes. Higher levels of MSLN-specific IgM and IgG antibodies were detected in the serum of vaccinated mice compared to that of control mice. Three vaccination regimens were tested: a prophylactic scheme that included vaccination before tumor induction and two therapeutic schemes involving early and late vaccination after tumor cell inoculation. MSLN nanovaccination inhibited KPC tumor progression and metastasis and induced higher CD8^+^ T cell infiltration in the tumor that developed in response to prophylactic and early therapeutic schedules but not in response to a later vaccination approach. Although the nanovaccine treatment elicited higher humoral and cellular antigen-specific responses in tumor-bearing mice for both vaccination strategies, the therapeutic vaccination also increased the expression of exhaustion markers in CD8^+^ T cells. **Conclusions**: Our results support the relevance of an MSLN-based nanovaccine as a new immunotherapy treatment for PDAC and propose an innovative method of vaccine delivery using NPs.

## 1. Introduction

Pancreatic ductal adenocarcinoma (PDAC) is characterized by abundant and dense stroma, composed of extracellular matrix proteins, fibroblasts, and infiltrated epithelial and suppressive immune cells [1]. Despite intense efforts to improve the clinical outcomes of this very aggressive cancer, patients have a dismal prognosis due to late diagnosis. With limited treatment options, standard approaches such as chemotherapy and radiotherapy show minimal effect on PDAC [2].

One emerging interest in cancer management is immunotherapy, which includes immune checkpoint inhibitors, oncolytic viruses, adoptive T cell therapies, and cancer vaccines [3,4]. While there have been advances in immunotherapy for some tumors, PDAC remains resistant [5]. Current treatments show minimal impact on patient survival, likely because PDAC evades immune surveillance. Additionally, PDAC tumors are considered immunologically “cold” due to limited T cell infiltration in their hypoxic and inaccessible tumor microenvironment [6].

Novel developments for therapeutic or preventive interventions against cancer include vaccines and adjuvants [7]. Based on the patient’s mutational profile or tumor-associated antigens (TAAs), peptide-based vaccines are designed to target the epitopes of these antigens that can induce an immune response. Mesothelin (MSLN) is a membrane-bound surface glycoprotein that is highly expressed in various solid tumors, including PDAC [8], and only in a limited number of healthy tissues, making it an attractive target for therapeutic strategies [9,10]. Previous studies have shown that MSLN affects pancreatic cell proliferation and migration and plays a role in tumor progression [11,12] and chemoresistance [13].

Nanoparticles (NPs) have been successfully used to improve the delivery of immunotherapeutic agents [14]. Immunostimulants can be added to vaccine formulations to enhance the activation of antigen-presenting cells (APCs), which are responsible for NP uptake, by targeting Toll-like receptors (TLRs) and boosting adaptive immune responses [15]. Resiquimod (R848) is a TLR 7/8 agonist, which induces the MyD88 immune signaling pathway and the secretion of transcriptional activator NF-κB, leading to the secretion of pro-inflammatory cytokines [16]. Polyinosinic-polycytidylic acid (poly I:C) binds TRL3, activating the TIR-domain-containing adapter-inducing IFN-β (TRIF) signaling pathway [17]. Both stimulants have shown potent antitumor activity and can be combined for use as vaccine adjuvants [18,19].

In this study, our nanovaccine was composed of an MSLN peptide and the adjuvants poly I:C and R848 encapsulated in PLGA–chitosan nanoparticles. Here, we provide evidence for the role of MSLN as an effective TAA that, when encapsulated with adjuvants into NPs, elicits strong immunogenicity. Our in vitro data show specific T cell activation in cells re-stimulated with the MSLN peptide. When a prophylactic vaccination approach and an early therapeutic scheme are applied, the MSLN nanovaccine results in increased humoral response and CD8^+^ T cell infiltration in the tumor as well as the inhibition of cancer progression. Overall, our data demonstrate the potential of an MSLN nanovaccine for the treatment of PDAC.

## 2. Materials and Methods

### 2.1. Animals and Ethics

Male pathogen-free C57BL/6 young-adult mice (12–15 weeks old) were obtained from the breeding facility of the Max-Planck-Institute for Multidisciplinary Sciences, Göttingen. The mice were fed a standard rodent chow (V1124-00, Sniff) with water ad libitum and experienced a 12 h light/dark cycle. All experiments were performed within the animal facility of the University Medicine Göttingen. All animal procedures were performed in compliance with the guidelines of the ARRIVE, the European Directive (2010/63/EU), and the German ethical laws and were approved by the administration of Lower Saxony, Germany (approval number G20.3527).

### 2.2. Peptide and NP Synthesis

We used an MSLN-derived synthetic long peptide (SLP) (palmitoyl-PLTVAEVQKLLGPHVKKALPLDLLLFLKKSLLFLLFSL-NH_2_) to generate the nanovaccine [20]. This 38-amino-acid peptide contains three MSLN epitopes, separated by KK, which are cathepsin-like cleavage sites. The three epitopes were predicted to elicit an immune response and were selected using public prediction servers [21]. A palmitoyl moiety was incorporated to increase their immunogenicity [22]. The multiple-epitope peptide was synthesized using a fluorenylmethoxycarbonyl (Fmoc)/tert-butyl (tBu) solid-phase peptide synthesis strategy and purified by reversed-phase chromatography. PLGA polymer (D, L-lactide/glycolide molar ratio of 50:50, M = 17,000 g/mol) and chitosan oligosaccharide lactate (Sigma Aldrich, St. Louis, MO, USA) were used for NP synthesis. Poly (lactic-co-glycolic acid) (PLGA)–chitosan nanoparticles (NPs) were synthesized using an oil-in-water emulsion, which was followed by solvent evaporation and extraction [23]. Specifically, 100 mg of PLGA powder was dissolved in 3 mL of dichloromethane. Depending on the type of NP, the solution was supplemented with 5 mg of poly I:C, 2 mg of R848, and/or 10 mg of MSLN peptide (prepared in 50% acetonitrile). This mixture was then added dropwise to 20 mL of a 2% (*w*/*v*) polyvinyl alcohol aqueous solution and emulsified using a sonicator for 60 cycles with 5 s rests between cycles. The solvents were left to evaporate overnight on a magnetic stirrer. The resulting NPs were collected by centrifugation (20,000 g for 30 min), redissolved in water, and subsequently added dropwise to 20 mL of a 1% homogenized chitosan oligosaccharide lactate solution. The mixture was stirred for 2 h at 4 °C. Finally, the chitosan-coated NPs were collected by lyophilization. Peptides and NPs were kept at −20 °C until dissolution with 50% acetonitrile or deionized water, respectively, and then stored at 4 °C for no more than one month. For the assessment of cellular uptake, the peptide was conjugated to a fluorophore (AZDye 568 NHS Ester, FP-1081, Vector Laboratories, Newark, CA, USA). NPs had a mean size of 355.5 nm (±23.8), determined by the dynamic light scattering (DLS) method.

### 2.3. PDAC Cells

The KPC cell line derived from tumors of KPC mice (Kras^LSL-G12D^, Trp53^−/−^, and PDX-1-Cre) was provided by Prof. Ellenrieder, University Medical Center Göttingen (UMG), Germany. KPC cells were cultured in DMEM supplemented with 10% fetal bovine serum, 1% penicillin and streptomycin (pen/strep), and 1% L-glutamine at 37 °C under a humidified atmosphere of 5% CO_2_.

### 2.4. Patient Samples

Human PDAC paraffin tissue sections were provided by Prof. Ströbel, collected and used according to the approval and regulations of the Ethics Committee of the UMG (Ethics approval #5/10/17 from 20 June 2023) and following the Declaration of Helsinki.

### 2.5. Vaccination

Mice were immunized by a subcutaneous (s.c.) injection on the left flank with 100 μL of either (i) empty NPs (NP control), (ii) NPs containing only the adjuvants poly I:C and R848 (2.5 µg and 3.75 µg per injection, respectively), (iii) NPs containing only MSLN peptide (50 µg per injection), or (iv) the nanovaccine, i.e., the NPs containing the MSLN peptide plus the adjuvants (vaccine), both at the same concentration as the control groups.

In addition, we evaluated the combination of MSLN nanovaccine and anti-PD-1 antibody. For this, mice received the same concentration of the nanovaccine and 100 µg of anti-PD-1 (BXC-BE0146, Biozol, Hamburg, Germany).

### 2.6. Bone Marrow-Derived Dendritic Cells (BMDCs)

For bone marrow isolation, the tibia and femur were removed and placed in ice-cold PBS. The bone marrow was flushed out and filtered using a 70 µm cell strainer. After centrifugation (300 g, 5 min), cells were resuspended in IMDM containing 10% FBS, 1% pen/strep, and 50 µM β-mercapto-ethanol and cultured for 14 days, with the medium changed every three days, at 37 °C and 5% CO_2_ in petri dishes in the presence of 20 ng/mL of recombinant mouse granulocyte-macrophage colony-stimulating factor (GM-CSF) for differentiation into BMDCs. For microscopy, 5 × 10^5^ cells/per well were plated on coverslips, while for flow cytometry, 1 × 10^5^ cells/per well were plated in a 96-well plate and incubated with the nanovaccine. Then, cells were fixed with 2% paraformaldehyde (PFA) after 4, 24, or 48 h of incubation, stained with Hoechst 33342 (Invitrogen, Waltham, MA, USA), and mounted on slides. For flow cytometry, cells were stimulated with the nanovaccine, MSLN peptide, or control NPs for 24 h and then harvested for further immune staining.

### 2.7. Ex Vivo Live-Cell Imaging

For the detection of the nanovaccine in lymph nodes (LNs), mice were vaccinated and euthanized after 4, 24, or 48 h. Their left inguinal LNs were excised for live-cell imaging, placed on uncoated 35 mm cell culture dishes (ibidi GmbH, Gräfelfing, Germany), covered entirely with 1% agarose–DMEM, and imaged by confocal microscopy using a Zeiss LSM880 microscope under live-cell imaging conditions (37 °C, 5% CO_2_). The AZDye™ 568 NHS Ester conjugated to the nanovaccine was excited at 561 nm and the autofluorescence of the tissue was determined at 488 nm.

### 2.8. Clearing and Multi-Photon Microscopy

For 3D analysis, entire LNs were fixed overnight with 4% PFA, then dehydrated in increasing ethanol concentrations (30%, 50%, 70%, 90%, 100%) for 4 h each, followed by an overnight incubation with 100% ethanol. Samples were cleared overnight with benzoic acid: benzyl benzoate (BABB, Sigma Aldrich, St. Louis, MO, USA) at a 1:2 ratio and kept in BABB at room temperature (RT) until imaging. 

Label-free second-harmonic generation (SHG) and fluorescence images were acquired using an upright TriM Scope Matrix multiphoton microscope (Miltenyi Biotec, Bergisch Gladbach, Germany) equipped with a wavelength-tunable laser source (1300–1700 nm, CRONUS-3P, Light Conversion, Vilnius, Lithuania). The incident laser power under the objective was ≈ 5 mW at 1300 nm. Images were acquired using a 25x Olympus (NA 1.05) water immersion objective. The backscattered fluorescence light was detected using photomultiplier tubes (PMT, Hamamatsu, Shizuoka, Japan). The SHG forward-scattered emitted light gathered by a 1.4 NA condenser lens under the stage was detected at a PMT in transmission position.

### 2.9. Preparation of Blood, Spleen, and Lymph Nodes

Blood was collected intracardially right after sacrifice. A 100 µL sample was mixed 1:1 with PBS for flow cytometry, while the rest was centrifuged at 1000× *g* for 30 min to obtain serum. Spleens and inguinal lymph nodes were removed and mechanically processed into single-cell suspensions. Red blood cells were eliminated using erythrocyte lysis buffer for 5 min at room temperature, and pellets were resuspended in RPMI 1640 medium.

### 2.10. Killing Assay

Splenocytes were stimulated with 20 µg/mL of MSLN peptide for 48 h. Cells were seeded in co-culture with KPC cells at a ratio of 20:1 (effector/target) in a 96-well plate for 48 h. Cell viability was evaluated by MTS assay according to the manufacturer’s protocol (Promega, Madison, WI, USA). The percentage of MSLN-induced cytotoxicity in the co-culture was calculated using the following equation: 100 − ([absorbance of co-culture wells − absorbance of effector cells]/absorbance of target cells × 100). In addition, IFN-γ levels were analyzed in the supernatant at the endpoint by ELISA.

### 2.11. Syngeneic Orthotopic PDAC Mouse Model and Tumor Preparation

The orthotopic tumor cell implantation followed a previously established protocol [24]. In summary, mice were anesthetized, a small midline incision was made to expose the pancreas, and 5 × 10^4^ KPC cells were injected into the pancreatic head in 15 µL of PBS. Tumor growth was assessed weekly by ultrasound using a VisualSonics Vevo 2100 device (FUJIFILM, Minato, Japan). Mice were euthanized, blood was extracted by cardiac puncture, the pancreatic tumors were excised and their sizes measured with a caliper, and the abdominal organs were excised and inspected for macroscopic metastases. One half of the tumor sample was fixed overnight in 4% PFA and embedded in paraffin. The other half was used for flow cytometry analysis by digesting small tumor pieces with an enzyme cocktail at 37 °C using the mouse Tumor Dissociation kit (Miltenyi Biotec) to obtain a single cell suspension, which was applied to a 70 µm cell strainer and resuspended with DMEM for further staining.

### 2.12. Flow Cytometry

Cells were stained for 30 min with the viability dye Zombie UV, followed by staining in the presence of Fc block (CD16/32) and mouse monoclonal antibodies as indicated in Supplementary Table S1. For the intracellular staining of splenocytes, cells were stimulated overnight with 10 µg/mL of MSLN peptide, followed by incubation with Brefeldin A (1:1000, Biolegend, Amsterdam, The Netherlands) for 4 h. Splenocytes were labeled with Zombie UV dye and surface antibodies, then fixed, permeabilized, and stained for intracellular interferon-γ (IFN-γ) expression at 4 °C in the dark. Samples were acquired using an LSRFortessa X-20 flow cytometer (BD Biosciences, Franklin Lakes, NJ, USA), and data analysis was performed with FlowJo software (version 10.8). The gating strategies for each panel are described in Supplementary Figures S2–S5.

### 2.13. Histology and Immunohistochemistry

Paraffin tissue sections of 4 μm were subjected to immunohistochemistry, as described previously [25]. Slides were incubated overnight with polyclonal rabbit antibody anti-MSLN (1:250, LS-C407883, LSBio, Seattle, WA, USA) for murine KPC tumor samples and with monoclonal mouse antibody anti-MSLN (1:200, ab236546, Abcam, Cambridge, UK) for human PDAC samples, followed by 1 h incubation with horseradish peroxidase (HRP)-conjugated goat anti-rabbit or anti-mouse secondary antibody, counterstaining of nuclei with hematoxylin, and mounting on slides. Images were acquired by an Axiovert 200 M microscope (Zeiss, Jena, Germany) equipped with an AxioCamHR camera.

### 2.14. Immunofluorescence

After fixation with 4% PFA, KPC cells were permeabilized, blocked with fish serum (37527, Invitrogen), and incubated with polyclonal rabbit antibody anti-MSLN (1:100). Cells were then incubated with a goat anti-rabbit secondary antibody conjugated with Alexa Fluor 647 (1:200, a21244, Invitrogen). Tumor tissue slides were subjected to antigen retrieval (pH 9.0), blocked with fish serum, and incubated overnight with rabbit primary monoclonal antibody against CD8b (1:500, ab228965, Sigma) or Granzyme B (1:350, 17215, Cell Signalling, Leiden, The Netherlands), followed by incubation with a goat anti-rabbit secondary antibody conjugated with Alexa Fluor 647 (1:200). Nuclei were counterstained with Hoechst (33342, Invitrogen) and mounted with coverslips. Images were acquired at a Leica SP8 confocal microscope, using a 20× objective (24 images per tumor section). Images were analyzed by Fiji ImageJ 1.54 to quantify the number of positive cells.

### 2.15. Enzyme-Linked Immunosorbent Assay (ELISA)

A standard sandwich ELISA was conducted to detect IFN-γ levels using a mouse Kit 88-7314-88 (Invitrogen). In summary, a 96-well plate was coated overnight at 4 °C with the capture antibody in coating buffer. After a 1 h blocking step, samples and standards were added and incubated for 2 h at room temperature. The plate was then treated with a biotinylated detection antibody for 1 h, followed by a 30 min incubation with streptavidin-peroxidase and finally with 3, 3′, 5, 5′-Tetramethylbenzidine substrate for 10 min at room temperature. The reaction was terminated with 2N H_2_SO_4_, and absorbance was measured at 450 nm using a plate reader (BioTek, Waldbronn, Germany). A similar procedure was performed to detect anti-MSLN IgG/IgM in the serum. For this, the plate was coated overnight with 10 µg/mL MSLN peptide. After blocking and incubating with samples, the plate was incubated with HRP anti-rabbit IgG (410406, Biolegend) or IgM antibody (31456, Invitrogen) at 1:2000 for 1 h and incubated with the substrate, as for IFN-γ ELISA.

### 2.16. Western Blotting

Samples were separated using 4–12% SDS–PAGE gels and transferred onto nitrocellulose membranes. The blots were blocked and incubated with polyclonal rabbit anti-MSLN antibody (1:500, LS-C407883, LSBio) at 4 °C overnight. Thereafter, goat anti-rabbit HRP-conjugated secondary antibody (1:2000, Amersham, Amersham, UK) was added and the protein bands in the membrane were visualized with enhanced chemiluminescence substrate. β-actin was used as a loading control (1:1000, Sigma-Aldrich, Darmstadt, Germany).

### 2.17. Statistics

Statistical analysis was carried out using GraphPad Prism V10. Data are expressed as mean ± SD. For multiple comparisons, unpaired Student’s *t*-test or one- or two-way ANOVA followed by Sidak’s post hoc test were applied. A *p*-value of <0.05 was considered statistically significant.

## 3. Results

As a potential target in the nanovaccine approach, we verified the expression of the tumor antigen MSLN in murine PDAC KPC cells and tumor samples from both PDAC patients and mice (Supplementary Figure S1), confirming that MSLN represents a suitable antigen target for vaccination.

### 3.1. MSLN Nanovaccine Induces DC Maturation After Internalization and Localizes in Draining LNs After Subcutaneous Inoculation

**Figure 1:**
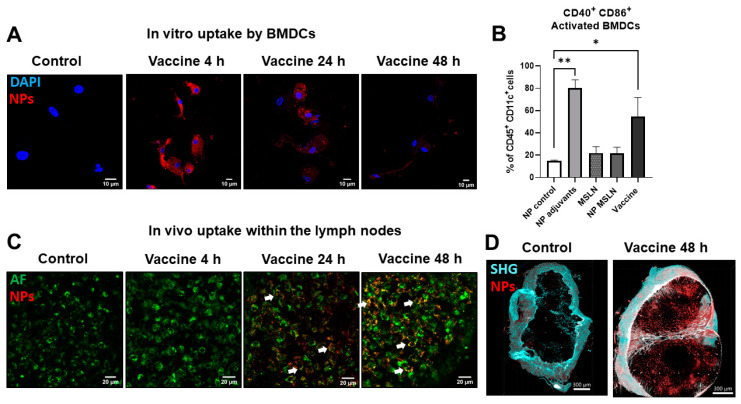
Nanovaccine uptake by bone marrow-derived dendritic cells (BMDCs) in vitro and by lymph node cells in vivo. (**A**) Representative images of BMDCs showing positive signals derived from the AZDye™ 568-conjugated nanovaccine (in red) after 4, 24, and 48 h of incubation, with the highest in vitro uptake at 4 h. Nuclei were stained with Hoechst after fixation (shown in blue). In untreated BMDCs, no positive signal was found. (**B**) BMDCs showed higher activation after incubation with NP adjuvants or nanovaccine compared to control groups. (**C**) Representative images of LN tissue slices from mice immunized with the AZDye™ 568-conjugated nanovaccine and euthanized after 4, 24, and 48 h. Nanovaccine-derived signals were visible from 24 h after immunization (white arrows). Non-vaccinated mice did not show any nanovaccine-specific fluorescent signals within the LNs. Images were acquired with a resolution of 1024 × 1024 pixels with a 20× objective. (**D**) The 3D imaging of cleared LNs shows nanovaccine-derived fluorescent signals (red) 48 h after mice were immunized. Label-free second-harmonic generation (SHG) of the collagen fibers in LN capsules reveals a 2.7-fold increase in the LN size from vaccinated mice in comparison to those from non-vaccinated mice. Data are presented as mean ± SD. One-way ANOVA, followed by Sidak’s multiple comparisons, n = 3–4 per group. * *p* < 0.05, ** *p* < 0.01. AF: autofluorescence, NP: nanovaccine.

The cellular uptake of the nanovaccine by APCs was investigated in CD11c^+^-positive BMDCs (confirmed by flow cytometry). After 4 h of incubation with the nanovaccine, a strong positive fluorescent signal was observed around the nuclei of the cells, which persisted for 48 h, demonstrating that the nanovaccine was efficiently internalized by the BMDCs. Non-stimulated BMDCs did not take up the nanovaccine and therefore did not show any positive fluorescent signals (Figure 1A). Incubation with NPs containing the adjuvants poly I:C and R848 (with or without the MSLN peptide) resulted in the enhanced expression of the activation markers CD40 and CD86 by BMDCs (Figure 1B), indicating the importance of the adjuvants in the vaccine composition. The NP control or MSLN peptide alone failed to do so. The in vivo uptake of the nanovaccine in the lymph nodes (LNs) was then investigated. To this end, mice were immunized subcutaneously with the nanovaccine, and 4, 24, and 48 h later, inguinal LNs were collected. We expected an accumulation of NPs in the draining LNs as a result of APC migration [26,27]. Internalized nanovaccine was visible inside the LN tissue at 24 and 48 h after the nanovaccine s.c. injection (Figure 1C). The 3D volume projections of cleared LNs show the label-free second-harmonic generation (SHG) signals of the collagen fibers in LN capsules (Figure 1D, cyan signals) and the presence of the nanovaccine at the border and in the central areas of the LN (Figure 1D, red signals), revealing the internalization of NPs in the LN cells of vaccinated mice and the absence of these in non-vaccinated mice (Figure 1D).

### 3.2. MSLN Nanovaccine Induces Cellular and Humoral Response In Vitro

**Figure 2:**
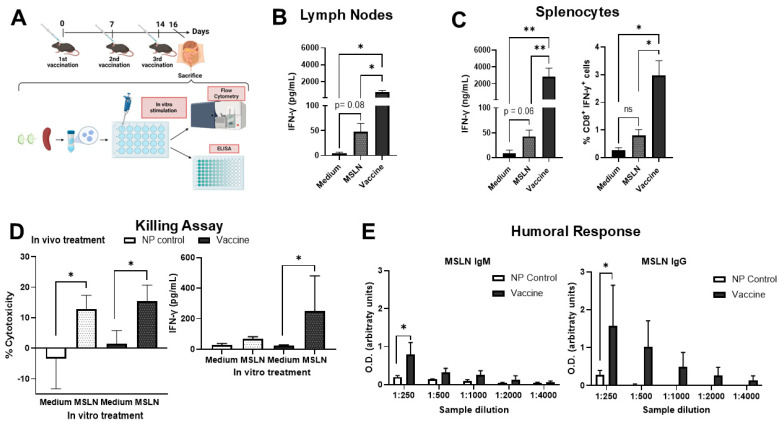
In vitro immunogenic response of mesothelin nanovaccine. (**A**) Mice were immunized three times with the nanovaccine and euthanized two days after the last booster. Cells from LNs and spleens were isolated and re-stimulated either with MSLN peptide or nanovaccine (10 µg/mL). (**B**) In the LNs, IFN-γ secretion was higher in nanovaccine-stimulated cells compared to control groups. (**C**) Similarly, nanovaccine-stimulated splenocytes showed higher IFN-γ production than unstimulated or peptide-stimulated cells, measured by ELISA (**left**) and intracellular staining (**right**). (**D**) For the killing assay, splenocytes from mice immunized with the nanovaccine or NP control were re-stimulated for 48 h with MSLN peptide (20 µg/mL) and then co-cultured with KPC cells for two days. MSLN-activated splenocytes elicited higher cytotoxicity in comparison to unstimulated splenocytes (**left panel**). Increased levels of IFN-γ were found in the supernatant of the co-cultures with MSLN-peptide-re-stimulated splenocytes isolated from vaccinated mice in comparison to unstimulated splenocytes from vaccinated mice (**right panel**). (**E**) IgM and IgG antibodies against MSLN were found to be elevated in the serum of nanovaccine-immunized mice compared to NP control mice. Data are presented as mean ± SD. One-way ANOVA followed by Sidak’s multiple comparisons was performed in (**B**,**C**), n = 3–7 per group; two-way ANOVA followed by Sidak’s multiple comparisons was performed in (**D**), n = 3 per group; unpaired *t*-test was performed in (**E**), n = 3 per group. * *p* < 0.05, ** *p* < 0.01, ns = not significant.

For the evaluation of nanovaccine immunogenicity, mice were immunized s.c. three times every week and euthanized two days after the last boost (Figure 2A). An MSLN-specific cellular response was investigated by in vitro re-stimulation of isolated LN cells and splenocytes with the MSLN peptide or the whole nanovaccine. Compared to unstimulated cells, increased secretion of IFN-γ was found in both LN cells and splenocytes after nanovaccine re-stimulation (Figure 2B,C, left). Although it did not reach significance, re-stimulation with the free MSLN peptide elicited an increase in IFN-γ production by LN cells and splenocytes. To further confirm this finding, intracellular flow cytometry was performed. The nanovaccine and, to a lesser extent, MSLN peptide induced IFN-γ production by CD8^+^ T cells (Figure 2C, right).

We further assessed the antigen-specific cytotoxicity of splenocytes to KPC target cells via a killing assay. For this, splenocytes from mice that were immunized with either nanovaccine or the NP control were re-stimulated with MSLN peptide. Then, splenocytes were co-cultured with KPC cells for 48 h and the percentage of viable tumor cells was measured by MTS assay. MSLN peptide stimulation elicited an increase in KPC cell death in comparison to co-culture with unstimulated cells (Figure 2D, left panel). Surprisingly, we observed MSLN-induced cytotoxicity with splenocytes from both groups of mice, those immunized with the nanovaccines and the NP control. Additionally, we observed an increased level of IFN-γ in the supernatant of the co-culture with KPC cells and splenocytes isolated from vaccinated mice and re-stimulated with MSLN peptide (Figure 2D, right panel) in comparison to unstimulated splenocytes from vaccinated mice.

Humoral response against MSLN was evaluated by ELISA by determining the levels of antibodies in the serum of vaccinated mice. Our data showed an increase in IgM and IgG antibodies in mice immunized with the nanovaccines in comparison to NP control mice (Figure 2E). Overall, our results demonstrated that the MSLN nanovaccine elicited both cellular and humoral immune responses and can thus be considered as a potential candidate for anti-tumor immune therapy in vivo.

### 3.3. Prophylactic Treatment with MSLN Nanovaccine Delays Tumor Progression

**Figure 3:**
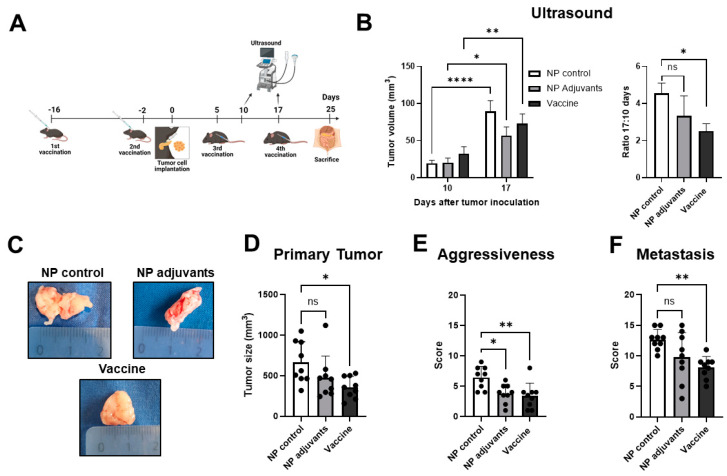
Anti-tumor efficacy of MSLN prophylactic vaccination. (**A**) Mice were immunized twice either with the nanovaccine or controls before orthotopic implantation of 5 × 10^4^ KPC cells. Then, mice received two boosters and were euthanized 8 days after the last booster. (**B**) Tumor growth was monitored by ultrasound 10 and 17 days after cell implantation. The tumor growth ratio, calculated by dividing the tumor size on day 17 by the size on day 10, was higher in the NP control group compared to the nanovaccine group. (**C**) Representative images of KPC tumors from the different treatment groups on day 25 are presented. In comparison to the NP control group, vaccinated mice showed significant differences, including smaller primary tumors (**D**), lower scores in aggressiveness (**E**), and less metastasis (**F**). Data are presented as mean ± SD. Two-way ANOVA followed by Sidak’s multiple comparisons was performed in (**B**,**left**), n = 9 per group; one-way ANOVA followed by Sidak’s multiple comparisons was performed in (**B**,**right**) and (**D**), n = 9 per group. * *p* < 0.05, ** *p* < 0.01, **** *p* < 0.0001, ns = not significant.

We then tested the potential efficacy of the MSLN nanovaccine in inhibiting tumor growth, using a prophylactic vaccination approach. For this purpose, mice were immunized two times before the orthotopic implantation of KPC cells. Mice received nanovaccine boosters 5 and 17 days after implantation and were euthanized 8 days after the last vaccination (Figure 3A). The vaccination was well tolerated by all mice with no visible side effects, such as weight loss or skin rashes.

By ultrasound, we observed an increase in tumor volume over time in all treatment groups (Figure 3B, left panel). However, the ratio of tumor size on day 17 to tumor size on day 10 was significantly lower in mice that received the nanovaccine, indicating that the growth rate was lower relative to control (empty NP) mice (Figure 3B, right panel). We examined the size and morphology of the primary tumors, as well as the invasion of adjacent organs (Figure 3C). Tumors from vaccinated mice were well defined and encapsulated, with non-invasive borders, whereas the tumors from NP-control-treated mice appeared less delineated and invaded other organs. Additionally, vaccinated mice presented significantly smaller primary tumors (357.5 ± 132.9 mm^3^) than NP control mice (661.9 ± 255.5 mm^3^; Figure 3D). We developed an aggressiveness score, as PDAC is known to invade vital organs such as the stomach and duodenum, and its infiltration into these structures significantly impacts surgical resectability and worsens patient prognosis. Moreover, the presence of ascites further reflects the tumor’s aggressiveness and its potential clinical implications. Mice treated with the nanovaccine and NP adjuvants presented lower aggressiveness scores compared to controls (Figure 3E). Furthermore, MSLN vaccination decreased the occurrence of metastasis in the diaphragm, liver, kidneys, mesentery, and spleen when compared to unvaccinated mice (Figure 3F). Treatment with NP, containing only adjuvants, also led to a lower score for aggressiveness and induced smaller primary tumors. However, this did not reach significance when compared to untreated mice. The scores for aggressiveness and metastasis are indicated in Supplementary Tables S2 and S3, respectively.

### 3.4. Prophylactic Treatment with MSLN Nanovaccine Elicits an Immune Response in Tumor-Bearing Mice

**Figure 4:**
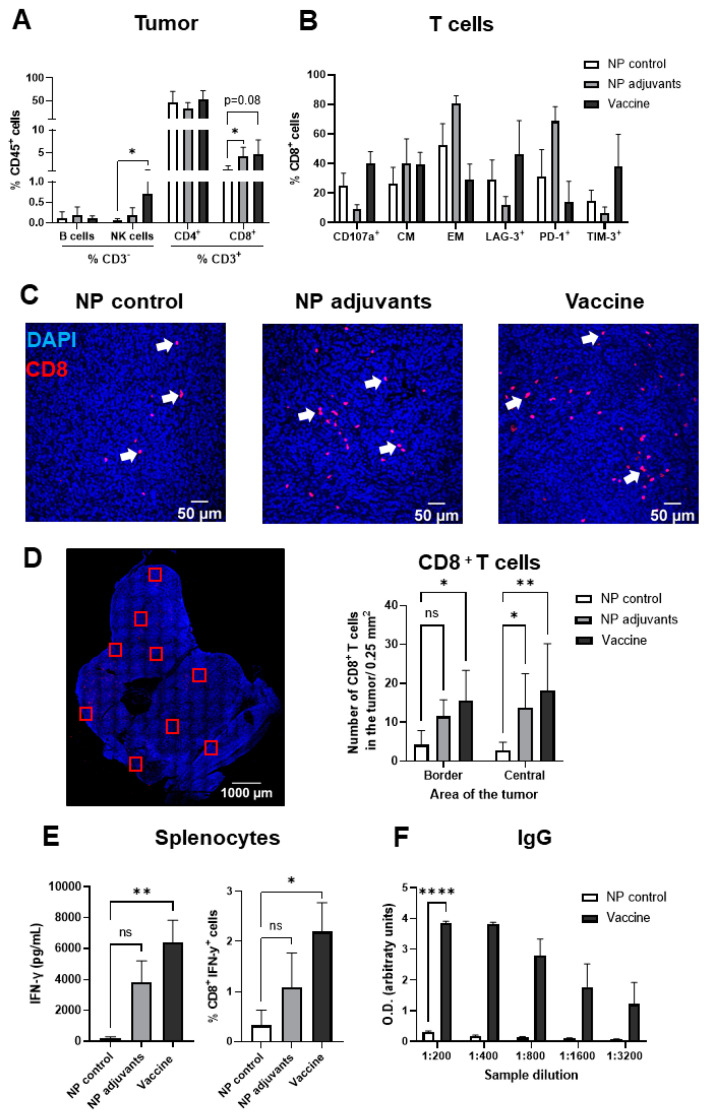
Prophylactic treatment with MSLN nanovaccine elicits an immune response. (**A**) Analysis of tumor samples by flow cytometry for subpopulations of lymphoid cells (B cells, natural killer (NK) cells, CD4^+^ and CD8^+^ T cells) revealed an increased number of NKs and CD8^+^ T cells in vaccinated mice. (**B**) The subpopulation of CD8^+^ T cells was assessed for degranulation marker (CD170a), markers for central memory cells (CMs; CD62L^+^ and CD44^+^), effector memory cells (EMs; CD62L^−^ and CD44^+^), and the exhaustion markers LAG-3, PD-1 and TIM-3. (**C**) Representative images of immunofluorescence-stained KPC tumors showing the infiltration of CD8^+^ T cells (red). Nuclei stained with Hoechst are shown in blue. White arrows point to CD8^+^ stainings. (**D**) ROIs were captured in the border and central area of the tumors using 20× magnification, as illustrated by the red squares. Quantitative analysis showed an increased number of infiltrated CD8^+^ T cells in the border and central areas of the tumors from vaccinated mice in comparison to tumors from the NP control group. Mice treated with NP adjuvants showed elevated numbers of CD8^+^ T cells in the central area of the tumors compared to NP control mice. (**E**) Splenocytes from tumor-bearing mice were isolated and re-stimulated with MSLN peptide (10 µg/mL). Note that cells from vaccinated mice presented the highest levels of IFN-γ production, evaluated by ELISA (**left**) and intracellular IFN-γ staining (**right**). (**F**) Serum IgG levels from vaccinated mice were elevated in comparison to NP control mice. Data are presented as mean ± SD. One-way ANOVA followed by Sidak’s multiple comparisons was performed in (**A**,**B**), n = 4–5 per group; in (**E**,**left**), n = 8–9 per group; in (**E**,**right**), n = 4–5 per group; and in (**F**), n = 3 per group. Two-way ANOVA followed by Sidak’s multiple comparisons was performed in (**D**), n = 6 per group. * *p* < 0.05, ** *p* < 0.01, **** *p* < 0.0001, ns = not significant.

Tumor samples were analyzed for lymphoid cell infiltration by flow cytometry. In vaccinated mice, there was an increase in the percentage of NK cells in comparison to NP controls (Figure 4A). Between the groups, we found a similar percentage of B and CD4^+^ T cell populations in the tumors. CD8^+^ T cell numbers were increased in the tumors from vaccinated mice and mice that received NP adjuvants, with the latter reaching significance. In comparison to mice treated with the NP control, tumors from vaccinated mice presented increased numbers of CD8^+^ cells expressing degranulation (CD107a) and exhaustion (LAG-3, TIM-3) markers. However, due to high variability within the group, the difference did not reach significance. In the same way, there was a tendency in the tumors from mice that received NP adjuvants to show increased numbers of effector memory (EM; CD62L^−^ and CD44^+^) cells and increased expression of PD-1 in comparison to the NP control group (Figure 4B). There was no difference in the number of central memory cells (CMs; CD62L^+^ and CD44^+^) between the groups. Through immunofluorescence, we further observed an increased infiltration of CD8^+^ cytotoxic T cells (CTLs) in the border and center of tumors from vaccinated mice in comparison to the control group (Figure 4C,D). The elevation of CTLs in the tumors of the NP adjuvant group was only significant in the central area of the tumor in comparison to NP group. Moreover, we evaluated the number of Granzyme B^+^ cells as an indication of CTL activity. We observed a higher number of Granzyme B^+^ cells in both the border and the central areas of the tumor from MSLN nanovaccinated mice compared to mice that received the NP control. (Supplementary Figure S6, left panel).

Splenocytes isolated from tumor-bearing mice were further stimulated in vitro with MSLN peptide for the assessment of specific T cell responses. Through ELISA (Figure 4E, left) and intracellular staining (Figure 4E, right), we found increased levels of IFN-γ in splenocytes from vaccinated mice, indicating higher T cell activation compared to the NP control. Furthermore, in vaccinated tumor-bearing mice, a higher quantity of IgG antibodies against MSLN was detected relative to mice treated with the NP control, suggesting an increased humoral response (Figure 4F).

### 3.5. Early Treatment with MSLN Vaccine Delays Tumor Progression

**Figure 5:**
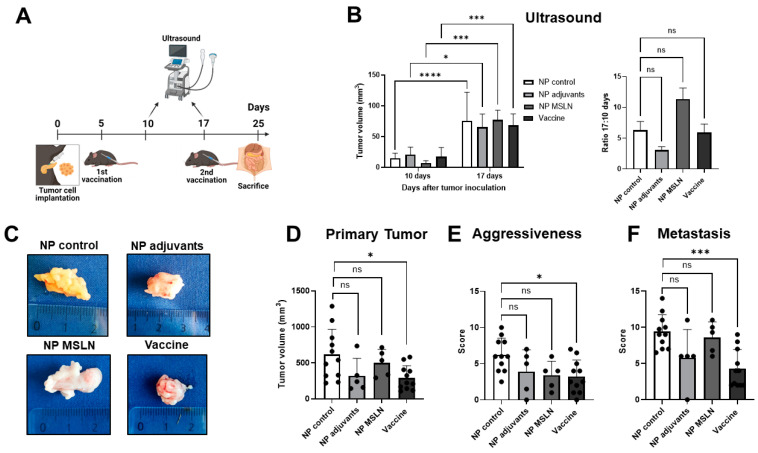
Anti-tumor efficacy of early therapeutic MSLN nanovaccination. (**A**) Mice were orthotopically implanted with 5 × 10^4^ KPC cells, immunized two times with the nanovaccine or controls, starting 5 days after cell injection, and euthanized 8 days after the last booster. (**B**) Tumor growth was monitored by ultrasound 10 and 17 days after cell implantation. Note that mice in all groups developed tumors and showed similar growth rates. (**C**) Representative images of KPC tumors from the different treatment groups on the day of sacrifice. Vaccinated mice showed significantly (**D**) smaller primary tumors, (**E**) lower aggressiveness scores, and (**F**) lower metastasis scores when compared to the NP control group. Data are presented as mean ± SD. Two-way ANOVA followed by Sidak’s multiple comparisons was performed in (**B**,**left**), n = 9 per group; one-way ANOVA followed by Sidak’s multiple comparisons was performed in (**B**,**right**) and (**D**), n = 5–11 per group. * *p* < 0.05, *** *p* < 0.001, **** *p* < 0.0001, ns = not significant.

We further tested the efficacy of the MSLN nanovaccine using an early therapeutic approach. For this purpose, mice were immunized twice only after tumor cell implantation (Figure 5A). Ultrasound measurements showed a similar tumor development over time across all treatment groups (Figure 5B). At the end of the experiment (day 25), similar to the prophylactic treatment, vaccinated mice had more encapsulated tumors, while tumors from NP-control-treated groups had a more invasive phenotype (Figure 5C). Primary tumors from vaccinated mice were significantly smaller (292.7 ± 169.1 mm^3^) than those from NP control mice (615.5 ± 352.8 mm^3^) (Figure 5D). Moreover, vaccinated mice were characterized by significantly lower aggressiveness and metastasis scores compared to the control group (Figure 5E,F). While treatment with NPs containing only adjuvants or only the MSLN peptide also showed a trend towards lower primary tumor size, aggressiveness, and metastasis scores in comparison to controls, these alterations were not significant.

### 3.6. Early Therapeutic Treatment with Nanovaccine Elicits an Immune Response in Tumor-Bearing Mice

**Figure 6:**
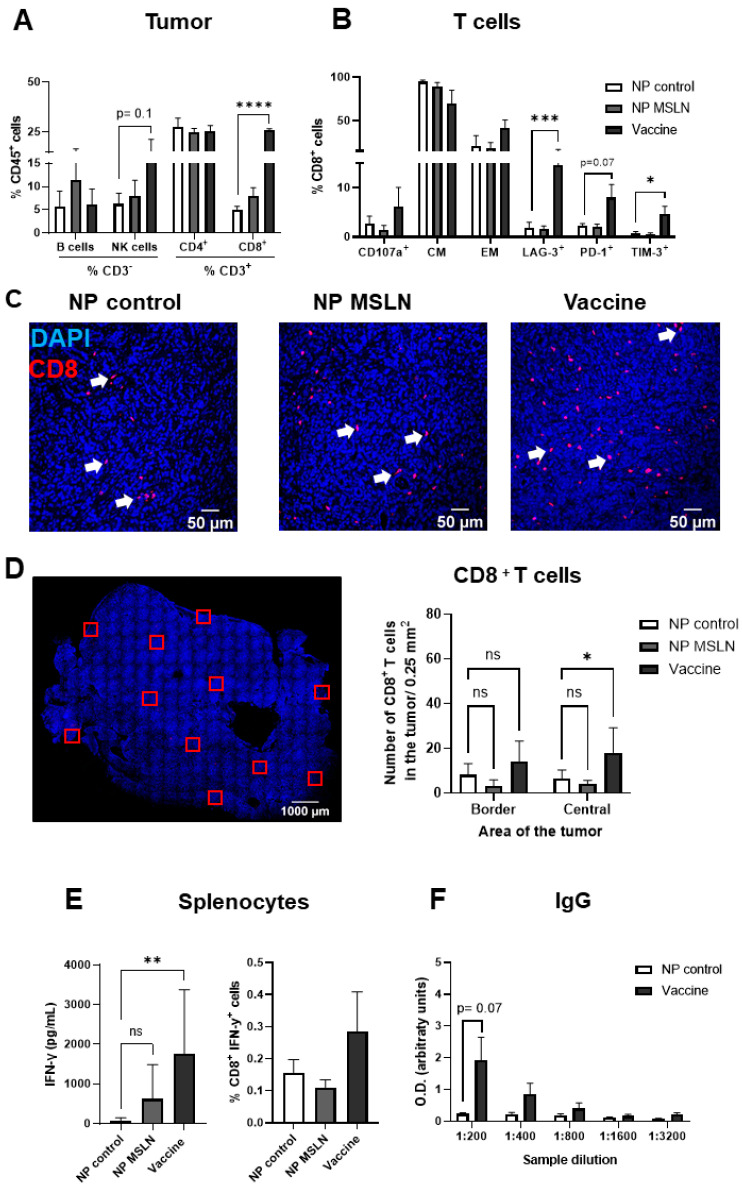
Early therapeutic treatment with MSLN nanovaccine elicits an immune response. (**A**) Analysis of tumor samples by flow cytometry for populations of lymphoid cells (B cells, natural killer (NK) cells, CD4^+,^ and CD8^+^ T cells) reveals a significantly increased number of CD8^+^ T cells in vaccinated mice compared to NP control mice. (**B**) Further analysis of the CD8^+^ T cell subpopulation showed elevated numbers of exhaustion-marker-expressing cells (LAG-3, PD-1, and TIM-3) but not of cells expressing the degranulation marker (CD170a), those expressing markers for central memory cells (CMs; CD62L^+^ and CD44^+^), or effector memory cells (EMs; CD62L^−^ and CD44^+^). (**C**) Representative images of immunofluorescence staining in KPC tumors showing the infiltration of CD8^+^ T cells (red). Nuclei stained with Hoechst are shown in blue. White arrows point to positive staining. (**D**) ROIs were captured at the border and in the central area of the tumors using 20× magnification, as illustrated by the red squares. Quantitative analysis showed an increased number of infiltrated CD8^+^ T cells in the central areas of the tumors from vaccinated mice, in comparison to the NP control group. (**E**) Splenocytes isolated from vaccinated tumor-bearing mice re-stimulated with MSLN peptide (10 µg/mL) presented the highest levels of IFN-γ production, evaluated by ELISA (**left**) and intracellular staining (**right**). (**F**) Serum IgG levels from vaccinated mice were elevated in comparison to NP control mice. Data are presented as mean ± SD. One-way ANOVA followed by Sidak’s multiple comparisons was performed in (**A**,**B**), n = 4–6 per group; in (**E**,**left**), n = 5–10 per group; in (**E**,**right**), n = 5 per group; and in (**F**), n = 3 per group. Two-way ANOVA followed by Sidak’s multiple comparisons was performed in (**D**), n = 5–6 per group. * *p* < 0.05, ** *p* < 0.01, *** *p* < 0.001, **** *p* < 0.0001, ns = not significant.

Similar to the prophylactic treatment, the early therapeutic vaccination led to a tendency towards an increased number of NK cells and a significantly higher percentage of tumor-infiltrated CD8^+^ T cells compared to that of mice from the control groups (Figure 6A). In addition, we found an elevated percentage of CD8^+^ T cells expressing the degranulation marker CD107a (not significant) and the exhaustion markers LAG-3, PD-1, and TIM-3 from vaccinated mice when compared to those from the NP control group (Figure 6B). The number of central memory cells (CMs; CD62L^+^ and CD44^+^) and effector memory cells (EMs; CD62L^−^ and CD44^+^) were unchanged. Quantitative immunofluorescence analysis showed the increased infiltration of CD8^+^ cytotoxic T cells in the center of tumors from mice treated with the nanovaccine in comparison to the NP control group (Figure 6C,D). Moreover, we evaluated the number of Granzyme B+ cells and found that mice treated with the MSLN nanovaccine showed an increased number of Granzyme B+ cells in the border areas of the tumors in comparison to the NP control group (Supplementary Figure S6, right panel). Furthermore, splenocytes from vaccinated tumor-bearing mice showed increased secretion of IFN-γ after re-stimulation with MSLN peptide (Figure 6E). Therapeutic vaccination with MSLN also induced higher levels of IgG antibodies in comparison to mice treated with the NP control (Figure 6F).

Due to the observed increase in exhaustion of tumor-infiltrated CD8^+^ T cells, we performed a combination study with the immune checkpoint inhibitor anti-PD1. This is a common combination strategy used in many vaccination studies. For this, mice were orthotopically implanted with KPC cells and divided into four groups according to the treatment: NP control, MSLN nanovaccine, anti-PD-1 antibody, and MSLN nanovaccine + anti-PD1 (n = 5–7 per group). Mice were treated with nanovaccine after 5 and 17 days of tumor cell implantation. In addition, mice received anti-PD-1 therapy twice per week, and all animals were sacrificed on the 22nd day. Mice that received only anti-PD-1 therapy also showed a reduction in tumor size and less aggressiveness scores compared to the control group. However, the combination therapy of the MSLN nanovaccine and anti-PD-1 did not enhance the effect of the single therapies, resulting in smaller primary tumors than in NP-control-treated mice but similar tumor sizes to the single treatments (nanovaccine or anti-PD-1 alone). In addition, mice treated with the combination had aggressiveness and metastasis scores that were almost as high as those of NP controls (Supplementary Figure S7).

### 3.7. Late Treatment with MSLN Nanovaccine Does Not Impact Tumor Growth

**Figure 7:**
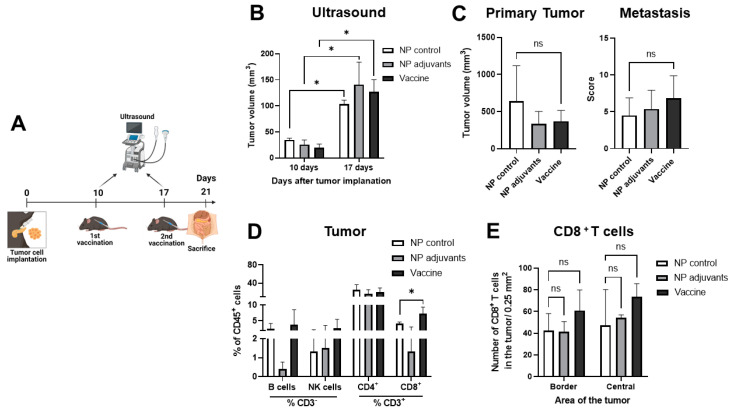
Low immune response to late therapeutic treatment with MSLN nanovaccine. (**A**) Mice were orthotopically implanted with 5 × 10^4^ KPC cells, immunized 10 and 17 days after cell implantation with the nanovaccines or controls, and euthanized 4 days after the last booster. (**B**) Tumor growth was monitored by ultrasound 10 and 17 days after cell implantation, showing similar tumor progression in mice from all treatment groups. (**C**) On the sacrifice day, we observed similar primary tumor sizes and comparable metastasis scores between groups. (**D**) Flow cytometry of tumor samples for populations of lymphoid cells (B cells, natural killer (NK) cells, CD4^+^, and CD8^+^ T cells) showed an increased number of CD8^+^ T cells in vaccinated mice. (**E**) Similar numbers of CD8^+^ T cell infiltration were detected by immunofluorescence at the border and in the central areas of the tumors from all mice. Data are presented as mean ± SD. Two-way ANOVA followed by Sidak’s multiple comparisons was performed in (**B**); one-way ANOVA followed by Sidak’s multiple comparisons was performed in (**C**–**E**), n = 3–4 per group. * *p* < 0.05, ns = not significant.

We then investigated whether MSLN vaccination at a later time point after cell implantation would still induce the same anti-tumor response that we observed at the early time point. Thus, mice received their first dose of the vaccine 10 days after tumor inoculation instead of 5 days after, with a booster one week later. Mice were euthanized 4 days after the last vaccination (Figure 7A). Ultrasound analysis showed similar tumor progression in mice from all treatment groups, suggesting that late vaccination did not change tumor growth kinetics (Figure 7B). Mice presented similar primary tumor sizes and comparable metastasis scores (Figure 7C). Flow cytometry analysis showed similar percentages of B, NK, and CD4^+^ T cells in the tumors of all treatment groups (Figure 7D). While we found an increase in CD8^+^ T cells in the vaccinated mice, in comparison to mice immunized with the NP control (Figure 7D), this increase could not be confirmed by immunofluorescence staining in tumor tissue sections (Figure 7E).

## 4. Discussion

Recent achievements in cancer immunotherapy underscore the advantages of using the immune system as a valuable tool for disease treatment. In our study, we combined two innovative approaches to induce a potent anti-tumor immune response: cancer vaccines and nanotechnology. We showed that a mesothelin peptide-based nanovaccine mediates a specific T cell response leading to KPC cell death in vitro. Moreover, both prophylactic and early therapeutic nanovaccination inhibit murine PDAC growth and metastasis while inducing CD8^+^ CTL infiltration in pancreatic tumors.

We demonstrated the overexpression of MSLN in murine KPC cells and KPC cell-induced tumors, in line with other reports. Due to its selective expression in tumors, MSLN has been proposed as a potential candidate for targeted immunotherapy by others. For our vaccine, we used a long peptide that comprises recognized immunogenic epitopes from human MSLN [28,29]. MSLN peptide, encapsulated in PLGA–chitosan NPs, was taken up by BMDCs after 4 h stimulation and was found in inguinal LN cells from 24 h after vaccination. In addition, we showed that the presence of the adjuvants poly I:C and R848 in the NPs is crucial for BMDC maturation. This is evidenced by the increased expression of the co-stimulatory molecules CD86 and CD40, which are necessary for the activation of naïve T cells into functional effector T cells [30]. The encapsulation of peptides and adjuvants into NPs facilitates the delivery of vaccine compounds, preserving them from rapid degradation and avoiding a local immune response, as reported by others [31].

We successfully demonstrated that the MSLN nanovaccine induces cellular and humoral immune responses. The in vitro re-stimulation with either MSLN peptide or the nanovaccine results in CD8^+^ T cell activation. In the case of cells re-stimulated with MSLN peptide, the increase in cytokine secretion indicates MSLN recognition by the adaptive immune system leading to an antigen-specific T cell activation. For the nanovaccine re-stimulation, the presence of adjuvants contributes to a higher immune response than for MSLN peptide-stimulated cells. It was expected that the adjuvants would potentiate the activation of T cells, as previously described by others [32,33] The MSLN-induced activity of CTLs is shown by an increased KPC cell death after co-culture with MSLN-stimulated splenocytes from vaccinated mice. Surprisingly, both splenocytes from immunized and non-immunized mice induced cytotoxicity to PDAC cells, suggesting that the in vivo priming of CTLs does not alter the T cell activation. Humoral immunity was evidenced by higher levels of IgM and IgG antibodies against the MSLN peptide in vaccinated mice compared to NP controls.

The MSLN nanovaccine efficiently prevents tumor growth through two vaccination approaches: prophylactic and early therapeutic. The first retards tumor growth observed by ultrasound and leads to the development of smaller tumors and less metastasis in comparison to mice that treated with the NP control. In the second approach, tumors are formed at a similar rate regardless of the treatment, but vaccinated mice still develop smaller primary tumors and metastasis than mice treated with control NPs, indicating that the priming of immune cells before tumor inoculation does not impact the tumor volume at the experiment’s endpoint. In addition, for both schedules, vaccinated mice presented encapsulated tumors, which are defined by the appearance of dense, fibrous connective tissue surrounding the tumor mass, associated with reduced invasiveness to adjacent organs and metastasis and thus a better prognosis [34]. However, the treatment was not effective when performed at a later stage after tumor development.

MSLN has also been a target for PDAC therapy by others using a wide range of methodologies. For example, a different MSLN peptide induced peptide-specific CTLs that were injected into mice with PDAC xenografts, leading to the inhibition of tumor growth [10]. In the genetically engineered KPC mouse model, affinity-enhanced T cell receptors against MSLN infiltrated the TME more efficiently, inducing cell death in PDAC tumors and prolonging the survival of KPC mice [35]. Furthermore, MSLN-directed chimeric antigen receptor T cells were shown to induce an effective anti-tumor response in preclinical models of PDAC [36,37].

In both prophylactic and therapeutic vaccination approaches, NP adjuvants only induced a non-significant reduction in tumor size compared to NP-control-injected mice. This indicates that while the adjuvants alone can modulate tumor progression, their combination with the MSLN peptide is necessary for effective tumor inhibition. Others have reported controversial results about the effect of adjuvants against PDAC. Similar to our findings, one report showed that a single treatment with R848 induced DCs and macrophage activation, but it failed to increase CTL or NK infiltration in the tumor or to eradicate PDAC tumors in an orthotopic mouse model [38]. On the other hand, R848 therapy resulted in smaller tumors and increased CTL infiltration in a different murine model of PDAC [39]. Likewise, the cytosolic delivery of poly I:C using polyethyleneimine [40] or intravenous injection [41] has been shown to induce KPC antitumor immunity in vivo, suggesting the efficacy of adjuvant-only therapy when using different methodologies.

Both prophylactic and early therapeutic approaches elicit an increase in NK cells and CD8^+^ T cell infiltration in the tumors. These cell types are known for their critical roles in anti-tumor immunity [24,42]. We hypothesize that our MSLN nanovaccine prevents tumor growth via the activation of CTLs. As demonstrated by us, the increased IFN-γ release by the re-stimulation of splenocytes (from tumor-bearing vaccinated mice) with the MSLN peptide supports the hypothesis that an antigen-specific T cell response against PDAC successfully leads to the prevention of disease progression. Although CD8+ T effector numbers and activity in the TME and spleen are strongly correlated with vaccine therapeutic benefit, formal confirmation of their critical involvement in treatment outcomes will need to be assessed in future targeted depletion assays.

Another possible mechanism for the efficacy of the MSLN nanovaccine against PDAC is the humoral response. Activated B cells can cause tumor apoptosis through antibody-dependent cellular cytotoxicity, an immune mechanism in which NK cells (bearing Fc receptors) recognize and eliminate antibody-coated tumor cells [43]. Since we observed increased MSLN-IgG antibodies and tumor-infiltrating NK cells in vaccinated mice, we cannot rule out the possibility that both B and NK cells contribute to the anti-tumor immune response elicited by MSLN vaccination. The functionality of such antibodies, however, was not evaluated in this study and could be the subject of an interesting analysis for the future to confirm the ability of the antibodies to bind MSLN-expressing tumor cells. Antibody-based therapy targeting MSLN has already shown promising results in preclinical and clinical studies [44,45]. Additionally, the blockage of MSLN with MUC16 by anti-MSLN antibodies can inhibit cancer cell expansion and metastasis [46] and exert therapeutic efficacy in mesotheliomas [47].

Interestingly, in our study, the early therapeutic nanovaccine scheme causes increased exhaustion in tumor-infiltrated T cells. T cells can become exhausted through persistent exposure to antigens and show increased apoptosis, low proliferation, and cytotoxic function [48]. We found higher numbers of exhausted T cells expressing several inhibitory receptors, including PD-1, CTLA-4, TIM-3, and LAG-3, that, upon binding to their ligands expressed in cancer cells, gradually reduce their effector function and proliferation [49], thus influencing the nanovaccine treatment outcome. It is known that in PDAC patients, co-expression of PD-1 and TIM-3 on T cells is correlated with worse prognosis [50], and single therapy with neutralizing antibodies against the inhibitory receptors has not been shown to improve clinical outcomes, indicating that multiple mechanisms contribute to the induction of exhausted T cells [51]. Notably, a combination therapy with MSLN nanovaccine and anti-PD1 antibody did not lead to a better anti-tumor effect than the single therapies and to higher aggressiveness and metastasis scores. We hypothesize that the combination treatment could exacerbate immune cell activation and lead to a number of unintended effects, such as hyperinflammatory response, overactivation of innate immunity, and immune checkpoint dysregulation.

## 5. Conclusions

Our findings suggest that the MSLN nanovaccine holds promise as a therapeutic target for PDAC treatment and could be further explored for translation into clinical settings.

## Data Availability

The data presented in this study are available on request from the corresponding author.

## Supplementary Materials

The following supporting information can be downloaded at: https://www.mdpi.com/article/10.3390/vaccines13030314/s1, Supplementary Table S1: List of antibodies used for flow cytometry; Supplementary Table S2: Scoring system for tumor aggressiveness; Supplementary Table S3: Scoring system for metastases; Supplementary Figure S1: Mesothelin overexpression in KPC cells as well as in human and murine tumor samples; Supplementary Figure S2: Gating strategy for flow cytometry of activated bone marrow-derived dendritic cells (BMDCs); Supplementary Figure S3: Gating strategy for flow cytometry of splenocytes; Supplementary Figure S4: Gating strategy for flow cytometry of the lymphoid cells; Supplementary Figure S5: Gating strategy for flow cytometry of T cells; Supplementary Figure S6: Granzyme B+ cells in the tumors; Supplementary Figure S7: Combination treatment with MSLN nanovaccine and anti-PD-1.

## References

[1] Liot S., Balas J., Aubert A., Prigent L., Mercier-Gouy P., Verrier B., Bertolino P., Hennino A., Valcourt U., Lambert E. (2021). Stroma Involvement in Pancreatic Ductal Adenocarcinoma: An Overview Focusing on Extracellular Matrix Proteins. Front. Immunol..

[2] Ryan D.P., Hong T.S., Bardeesy N. (2014). Pancreatic Adenocarcinoma. N. Engl. J. Med..

[3] Timmer F.E.F., Geboers B., Nieuwenhuizen S., Dijkstra M., Schouten E.A.C., Puijk R.S., de Vries J.J.J., Petrousjka van den Tol M., Bruynzeel A.M.E., Streppel M.M. (2021). Pancreatic Cancer and Immunotherapy: A Clinical Overview. Cancers.

[4] Chouari T., La Costa F.S., Merali N., Jessel M.D., Sivakumar S., Annels N., Frampton A.E. (2023). Advances in Immunotherapeutics in Pancreatic Ductal Adenocarcinoma. Cancers.

[5] Balachandran V.P., Beatty G.L., Dougan S.K. (2019). Broadening the Impact of Immunotherapy to Pancreatic Cancer: Challenges and Opportunities. Gastroenterology.

[6] Das M., Zhou X., Liu Y., Das A., Vincent B.G., Li J., Liu R., Huang L. (2020). Tumor Neoantigen Heterogeneity Impacts Bystander Immune Inhibition of Pancreatic Cancer Growth. Transl. Oncol..

[7] Donninger H., Li C., Eaton J.W., Yaddanapudi K. (2021). Cancer Vaccines: Promising Therapeutics or an Unattainable Dream. Vaccines.

[8] Le K., Wang J., Zhang T., Guo Y., Chang H., Wang S., Zhu B. (2020). Overexpression of Mesothelin in Pancreatic Ductal Adenocarcinoma (PDAC). Int. J. Med. Sci..

[9] Nichetti F., Marra A., Corti F., Guidi A., Raimondi A., Prinzi N., de Braud F., Pusceddu S. (2018). The Role of Mesothelin as a Diagnostic and Therapeutic Target in Pancreatic Ductal Adenocarcinoma: A Comprehensive Review. Target. Oncol..

[10] Tsukagoshi M., Wada S., Hirono S., Yoshida S., Yada E., Sasada T., Shirabe K., Kuwano H., Yamaue H., Tsukagoshi M. (2018). Identification of a Novel HLA-A24-Restricted Cytotoxic T Lymphocyte Epitope Peptide Derived from Mesothelin in Pancreatic Cancer. Oncotarget.

[11] Bharadwaj U., Li M., Chen C., Yao Q. (2008). Mesothelin-Induced Pancreatic Cancer Cell Proliferation Involves Alteration of Cyclin E via Activation of Signal Transducer and Activator of Transcription Protein 3. Mol. Cancer Res..

[12] Li M., Bharadwaj U., Zhang R., Zhang S., Mu H., Fisher W.E., Brunicardi F.C., Chen C., Yao Q. (2008). Mesothelin Is a Malignant Factor and Therapeutic Vaccine Target for Pancreatic Cancer. Mol. Cancer Ther..

[13] Cheng W.F., Huang C.Y., Chang M.C., Hu Y.H., Chiang Y.C., Chen Y.L., Hsieh C.Y., Chen C.A. (2009). High Mesothelin Correlates with Chemoresistance and Poor Survival in Epithelial Ovarian Carcinoma. Br. J. Cancer.

[14] Elumalai K., Srinivasan S., Shanmugam A. (2024). Review of the Efficacy of Nanoparticle-Based Drug Delivery Systems for Cancer Treatment. Biomed. Technol..

[15] Zhao T., Cai Y., Jiang Y., He X., Wei Y., Yu Y., Tian X. (2023). Vaccine Adjuvants: Mechanisms and Platforms. Signal Transduct. Target. Ther..

[16] Zhou Y., Chen X., Cao Z., Li J., Long H., Wu Y., Zhang Z., Sun Y. (2021). R848 Is Involved in the Antibacterial Immune Response of Golden Pompano (Trachinotus Ovatus) Through TLR7/8-MyD88-NF-ΚB-Signaling Pathway. Front. Immunol..

[17] Matsumoto M., Seya T. (2008). TLR3: Interferon Induction by Double-Stranded RNA Including Poly(I:C). Adv. Drug Deliv. Rev..

[18] Toussi D.N., Massari P. (2014). Immune Adjuvant Effect of Molecularly-Defined Toll-Like Receptor Ligands. Vaccines.

[19] Sajadian A., Tabarraei A., Soleimanjahi H., Fotouhi F., Gorji A., Ghaemi A. (2014). Comparing the Effect of Toll-like Receptor Agonist Adjuvants on the Efficiency of a DNA Vaccine. Arch. Virol..

[20] Luna O.F., Perez Y.V., Ferrari D.P., Sayedipour S.S., Royo M., Acosta G.A., Cruz L.J., Alves F., Agner E., Sydnes M.O. (2024). Impact of N-Terminal PEGylation on Synthesis and Purification of Peptide-Based Cancer Epitopes for Pancreatic Ductal Adenocarcinoma (PDAC). ACS Omega.

[21] Rammensee H.G., Bachmann J., Emmerich N.P.N., Bachor O.A., Stevanović S. (1999). SYFPEITHI: Database for MHC Ligands and Peptide Motifs. Immunogenetics.

[22] Stolk D.A., Horrevorts S.K., Schetters S.T.T., Kruijssen L.J.W., Duinkerken S., Keuning E., Ambrosini M., Kalay H., van de Ven R., Garcia-Vallejo J.J. (2021). Palmitoylated Antigens for the Induction of Anti-Tumor CD8+ T Cells and Enhanced Tumor Recognition. Mol. Ther. Oncolytics.

[23] Cruz L.J., Tacken P.J., Rueda F., Domingo J.C., Albericio F., Figdor C.G. (2012). Targeting Nanoparticles to Dendritic Cells for Immunotherapy. Methods Enzymol..

[24] Ferrari D.P., Ramos-Gomes F., Alves F., Markus M.A. (2024). KPC-Luciferase-Expressing Cells Elicit an Anti-Tumor Immune Response in a Mouse Model of Pancreatic Cancer. Sci. Rep..

[25] Markus M.A., Ferrari D.P., Alves F., Ramos-Gomes F. (2023). Effect of Tissue Fixation on the Optical Properties of Structural Components Assessed by Non-Linear Microscopy Imaging. Biomed. Opt. Express.

[26] Reddy S.T., Van Der Vlies A.J., Simeoni E., Angeli V., Randolph G.J., O’Neil C.P., Lee L.K., Swartz M.A., Hubbell J.A. (2007). Exploiting Lymphatic Transport and Complement Activation in Nanoparticle Vaccines. Nat. Biotechnol..

[27] Shima F., Uto T., Akagi T., Baba M., Akashi M. (2013). Size Effect of Amphiphilic Poly(γ-Glutamic Acid) Nanoparticles on Cellular Uptake and Maturation of Dendritic Cells in Vivo. Acta Biomater..

[28] Thomas A.M., Santarsiero L.M., Lutz E.R., Armstrong T.D., Chen Y.C., Huang L.Q., Laheru D.A., Goggins M., Hruban R.H., Jaffee E.M. (2004). Mesothelin-Specific CD8+ T Cell Responses Provide Evidence of In Vivo Cross-Priming by Antigen-Presenting Cells in Vaccinated Pancreatic Cancer Patients. J. Exp. Med..

[29] Wang B., Kuroiwa J.M.Y., He L.Z., Charalambous A., Keler T., Steinman R.M. (2009). The Human Cancer Antigen Mesothelin Is More Efficiently Presented to the Mouse Immune System When Targeted to the DEC-205/CD205 Receptor on Dendritic Cells. Ann. N. Y Acad. Sci..

[30] Singh-Jasuja H., Emmerich N.P.N., Rammensee H.G. (2004). The Tübingen Approach: Identification, Selection, and Validation of Tumor-Associated HLA Peptides for Cancer Therapy. Cancer Immunol. Immunother..

[31] Fujita Y., Taguchi H. (2017). Nanoparticle-Based Peptide Vaccines. Micro and Nanotechnology in Vaccine Development.

[32] Tandel N., Patel D., Thakkar M., Shah J., Tyagi R.K., Dalai S.K. (2024). Poly(I:C) and R848 Ligands Show Better Adjuvanticity to Induce B and T Cell Responses against the Antigen(s). Heliyon.

[33] Hotz C., Treinies M., Mottas I., Rötzer L.C., Oberson A., Spagnuolo L., Perdicchio M., Spinetti T., Herbst T., Bourquin C. (2016). Reprogramming of TLR7 Signaling Enhances Antitumor NK and Cytotoxic T Cell Responses. Oncoimmunology.

[34] Jackson T.L., Byrne H.M. (2002). A Mechanical Model of Tumor Encapsulation and Transcapsular Spread. Math. Biosci..

[35] Stromnes I.M., Schmitt T.M., Hulbert A., Brockenbrough J.S., Nguyen H.N., Cuevas C., Dotson A.M., Tan X., Hotes J.L., Greenberg P.D. (2015). T Cells Engineered against a Native Antigen Can Surmount Immunologic and Physical Barriers to Treat Pancreatic Ductal Adenocarcinoma. Cancer Cell.

[36] He J., Zhang Z., Lv S., Liu X., Cui L., Jiang D., Zhang Q., Li L., Qin W., Jin H. (2018). Engineered CAR T Cells Targeting Mesothelin by PiggyBac Transposon System for the Treatment of Pancreatic Cancer. Cell Immunol..

[37] Sun Q., Zhou S., Zhao J., Deng C., Teng R., Zhao Y., Chen J., Dong J., Yin M., Bai Y. (2018). Engineered T Lymphocytes Eliminate Lung Metastases in Models of Pancreatic Cancer. Oncotarget.

[38] Ye J., Mills B.N., Qin S.S., Garrett-Larsen J., Murphy J.D., Uccello T.P., Han B.J., Vrooman T.G., Johnston C.J., Lord E.M. (2022). Toll-like Receptor 7/8 Agonist R848 Alters the Immune Tumor Microenvironment and Enhances SBRT-Induced Antitumor Efficacy in Murine Models of Pancreatic Cancer. J. Immunother. Cancer.

[39] Michaelis K.A., Norgard M.A., Zhu X., Levasseur P.R., Sivagnanam S., Liudahl S.M., Burfeind K.G., Olson B., Pelz K.R., Angeles Ramos D.M. (2019). The TLR7/8 Agonist R848 Remodels Tumor and Host Responses to Promote Survival in Pancreatic Cancer. Nat. Commun..

[40] Bhoopathi P., Kumar A., Pradhan A.K., Maji S., Mannangatti P., Windle J.J., Subler M.A., Zhang D., Vudatha V., Trevino J.G. (2023). Original Research: Cytoplasmic-Delivery of Polyinosine-Polycytidylic Acid Inhibits Pancreatic Cancer Progression Increasing Survival by Activating Stat1-CCL2-Mediated Immunity. J. Immunother. Cancer.

[41] Metzger P., Kirchleitner S.V., Kluge M., Koenig L.M., Hörth C., Rambuscheck C.A., Böhmer D., Ahlfeld J., Kobold S., Friedel C.C. (2019). Immunostimulatory RNA Leads to Functional Reprogramming of Myeloid-Derived Suppressor Cells in Pancreatic Cancer. J. Immunother. Cancer.

[42] Uzhachenko R.V., Shanker A. (2019). CD8+ T Lymphocyte and NK Cell Network: Circuitry in the Cytotoxic Domain of Immunity. Front. Immunol..

[43] Sautès-Fridman C., Petitprez F., Calderaro J., Fridman W.H. (2019). Tertiary Lymphoid Structures in the Era of Cancer Immunotherapy. Nat. Rev. Cancer.

[44] Hassan R., Bullock S., Premkumar A., Kreitman R.J., Kindler H., Willingham M.C., Pastan I. (2007). Phase I Study of SS1P, a Recombinant Anti-Mesothelin Immunotoxin given as a Bolus I.V. Infusion to Patients with Mesothelin-Expressing Mesothelioma, Ovarian, and Pancreatic Cancers. Clin. Cancer Res..

[45] Kelly R.J., Sharon E., Pastan I., Hassan R. (2012). Mesothelin-Targeted Agents in Clinical Trials and in Preclinical Development. Mol. Cancer Ther..

[46] Avula L.R., Rudloff M., El-Behaedi S., Arons D., Albalawy R., Chen X., Zhang X., Alewine C. (2020). Mesothelin Enhances Tumor Vascularity in Newly Forming Pancreatic Peritoneal Metastases. Mol. Cancer Res..

[47] Baldo P., Cecco S. (2017). Amatuximab and Novel Agents Targeting Mesothelin for Solid Tumors. Onco Targets Ther..

[48] Wherry E.J. (2011). T Cell Exhaustion. Nat. Immunol..

[49] Saka D., Gökalp M., Piyade B., Cevik N.C., Sever E.A., Unutmaz D., Ceyhan G.O., Demir I.E., Asimgil H. (2020). Mechanisms of T-Cell Exhaustion in Pancreatic Cancer. Cancers.

[50] Nakano M., Ito M., Tanaka R., Yamaguchi K., Ariyama H., Mitsugi K., Yoshihiro T., Ohmura H., Tsuruta N., Hanamura F. (2018). PD-1+ TIM-3+ T Cells in Malignant Ascites Predict Prognosis of Gastrointestinal Cancer. Cancer Sci..

[51] Goulart M.R., Stasinos K., Fincham R.E.A., Delvecchio F.R., Kocher H.M. (2021). T Cells in Pancreatic Cancer Stroma. World J. Gastroenterol..

