# Supplementary material for: Anti-Tumor Efficacy of a Mesothelin-Based Nanovaccine in a KPC Orthotopic Mouse Model of Pancreatic Cancer
Source: Vaccines (Basel). 2025 Mar 14;13(3):314. doi: 10.3390/vaccines13030314 (PMC11946167; doi:10.3390/vaccines13030314)
Supplement: Supplementary file 1 [file vaccines-13-00314-s001.zip › vaccines-3410041-supplementary.pdf]

Anti-Tumor Efficacy of a Mesothelin-Based Nanovaccine in a KPC Orthotopic Mouse Model of Pancreatic Cancer

Ferrari, D.P.[1], Çobanoglu, O.[2], Sayedipour, S.[3], Luna, O.[4], Ferkel, S. A. M.[1], Agorku, D.[5], Perez, Y.[6], Cruz, L. J. [3], Albericio, F.[4], Trottein, F.[2], Alves, F.[1,7,8], Markus, .A.[1], Ramos-Gomes, F.[1*]

Supplementary material

Supplementary Table S1. List of antibodies used for flow cytometry. For each panel, the list of antibodies for surface and intracellular staining is indicated.

Antigen	*Clone*	*Company*
**BMDCs Activation**		
CD45	30-F11	Biolegend
CD11c	N418	Biolegend
CD40	3/23	Biolegend
CD86	GL-1	Biolegend
**Splenocytes Activation**		
CD45	30-F11	Biolegend
CD3	17A2	Biolegend
CD8a	53-6.7	Biolegend
CD4	RM4-5	Biolegend
IFN-γ	XMG1.2	Biolegend
**Tumor Lymphoid**		
CD45	30-F11	Biolegend
CD3	17A2	Biolegend
CD8a	53-6.7	Biolegend
CD4	RM4-5	Biolegend
NK1.1	PK136	Biolegend
CD19	6D5	Biolegend
**T cell analysis**		
CD45	30-F11	Biolegend
CD3	17A2	Biolegend
CD8a	53-6.7	Biolegend
CD4	RM4-5	Biolegend
CD107a	1D4B	Miltenyi Biotec
CD44	REA664	Miltenyi Biotec
CD62L	MEL14-H2.100	Miltenyi Biotec
LAG-3	C9B7W	Miltenyi Biotec
TIM-3	REA602	Miltenyi Biotec
PD-1	REA802	Miltenyi Biotec

Supplementary Table S2. Scoring system for tumor aggressiveness. Score sheet for the assessment of aggressiveness of tumor by the degree of invasion into neighboring organs and tumor growth at the scar in the mice. *Size reflects the general size of mesentery metastasis, as sizes were similar within one mouse. The aggressiveness score was obtained based on the infiltration of the primary tumor into the duodenum and stomach, the presence of ascites, and the size of the tumor that developed at the site of the scar in the abdomen. Each animal was scored separately.

**Aggressiveness**					
Ascites	*No*	*Yes*			
score	0	3			
Tumor invasiveness to adjacent organs	*No*	*Only stomach*	*Only duodenum*	*Both*	
Score	0	1.5	*1.5*	*3*	
scar metastases size (mm3)	*0*	*1-300*	*301-599*	*600-999*	*>1000*
score	0	1	2	3	4

Supplementary Table S3. Scoring system for metastases. Score sheet for the assessment of macroscopic metastases present in different organs. The degree of metastatic spread was assessed by applying a metastasis score, calculated as the sum of the values for each organ. For each organ, a number from 0 - 4 was given according to the number of metastases macroscopically observed. For the mesentery, the size of the metastases was also taken into account as it differed from mouse to mouse. However, mesentery metastasis sizes were similar within one mouse. Final mesentery scores were therefore calculated by multiplying the size score with the count score.

**Metastases**					
Liver metastases (count)	*0*	*1*	*2 or 3*	*>3*	
score	0	1	2	3	
Diaphragm (area)	*0*	*30%*	*50%*	*>50%*	
score	0	1	2	3	
Mesentery metastases (count)	*0*	*1 to 3*	*4 to 7*	*8 to 11*	*>11*
score	0	1	2	3	4
Mesentery metastases (size)*	0	1mm	2mm	3mm	>3mm
score	*0*	*1*	*2*	*3*	*4*
Total mesentery score is: count x size					
Kidney metastases	*No*	*Yes*			
score	0	1			
Spleen metastases	*No*	*Yes*			
score	0	1			
Lungs metastases	*0*	*1*	*2 or 3*	*>3*	
score	0	1	2	3	

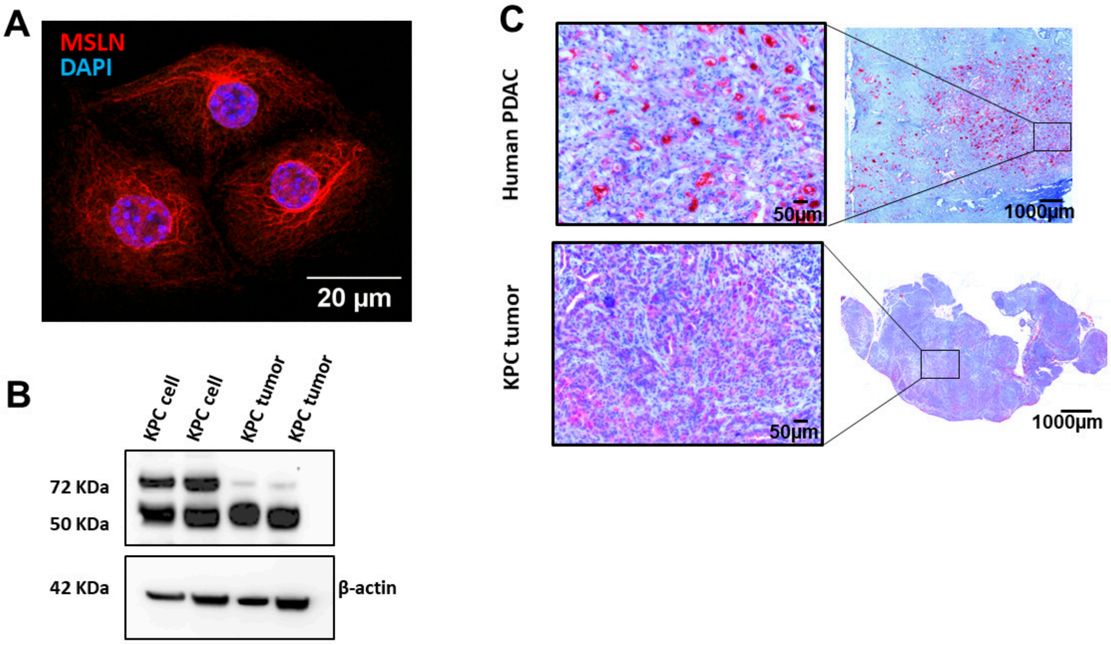

Supplementary Figure S1. Mesothelin overexpression in KPC cells as well as in human and murine tumor samples. (A) Positive staining of mesothelin (MSLN; in red) in KPC cells demonstrated by immunofluorescence. Nuclei are stained by Hoechst and shown in blue. (B) Positive MSLN protein expression in KPC cells was assessed by western blotting, showing bands at 72 kDa (its precursor form) and at ~50 kDa (cleaved form). β-actin was used as a loading control and showed bands at ~42 kDa. (C) Representative images of human PDAC and murine KPC tumor tissue sections showing MSLN positive expression in tumor cells by immunohistochemistry (overview, right and magnification, left).

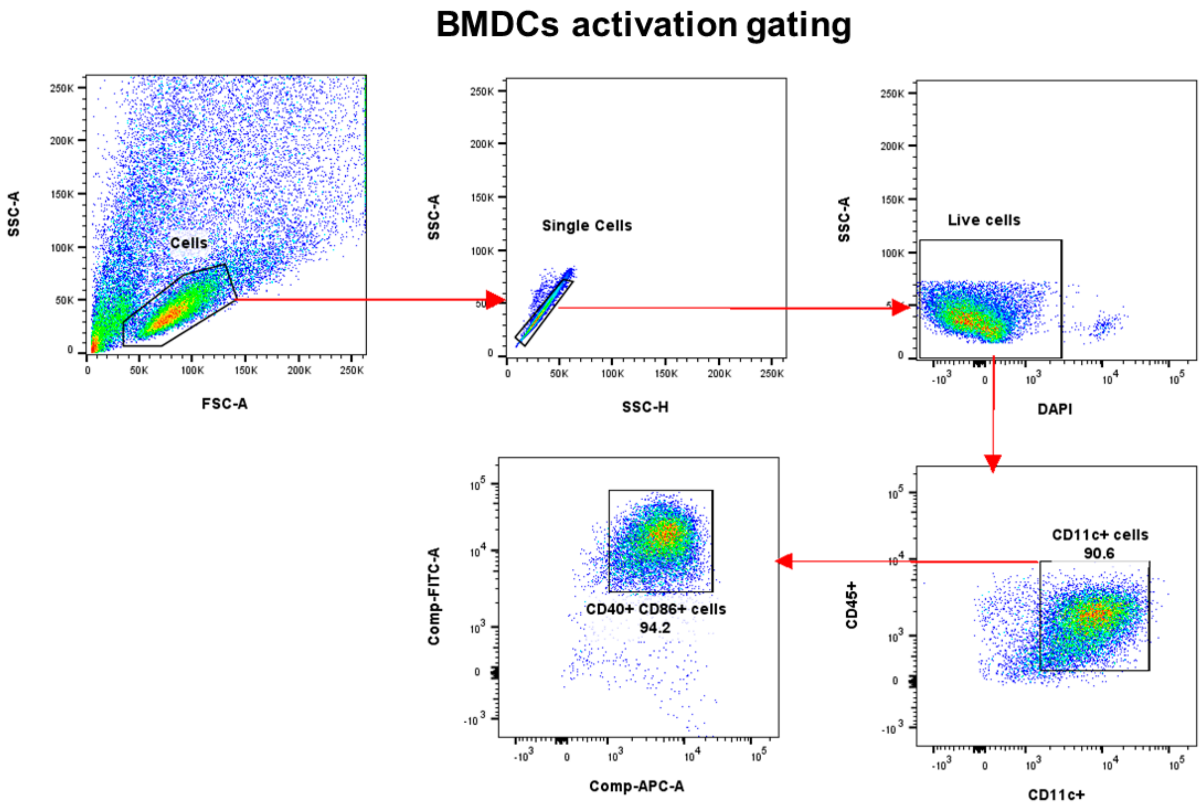

Supplementary Figure S2. Gating strategy for flow cytometry activated bone marrow-derived dendritic cells (BMDCs). Gating for the activation markers CD40 and CD86 in the BMDCs.

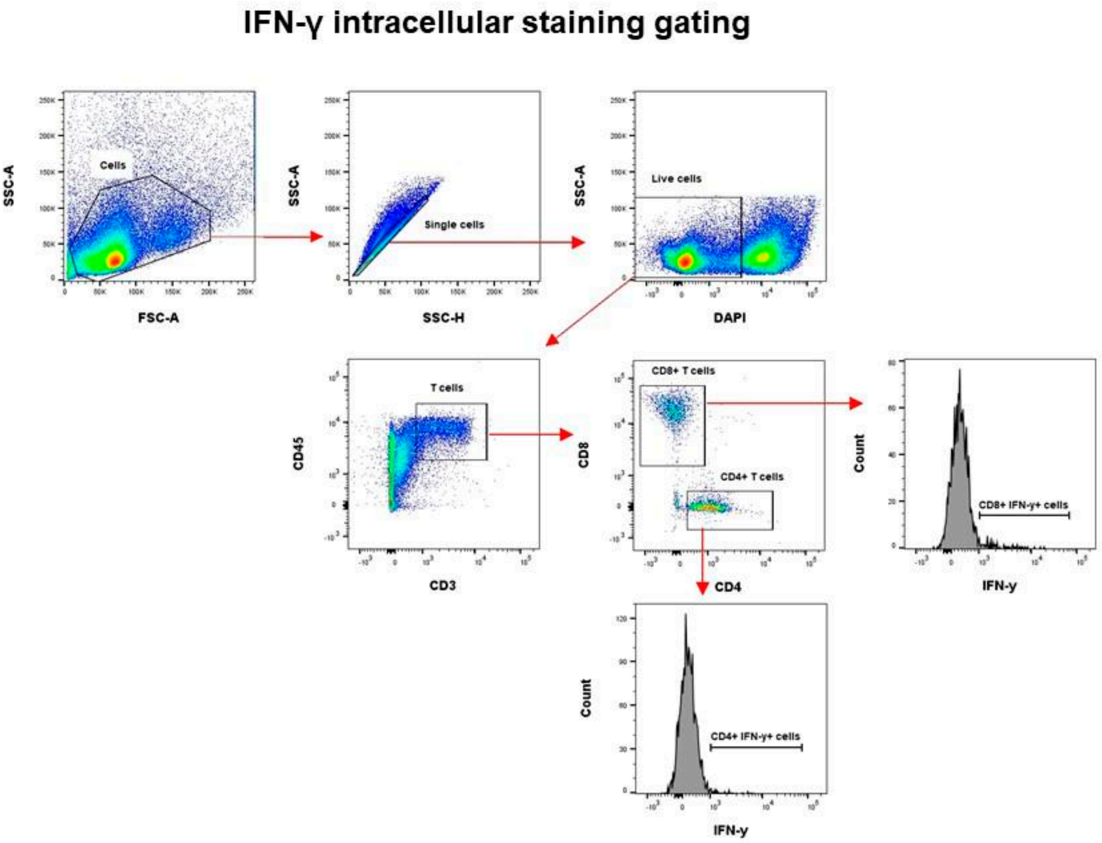

Supplementary Figure S3. Gating strategy for flow cytometry of splenocytes. Gating for IFN-γ^+ cells after intracellular staining.

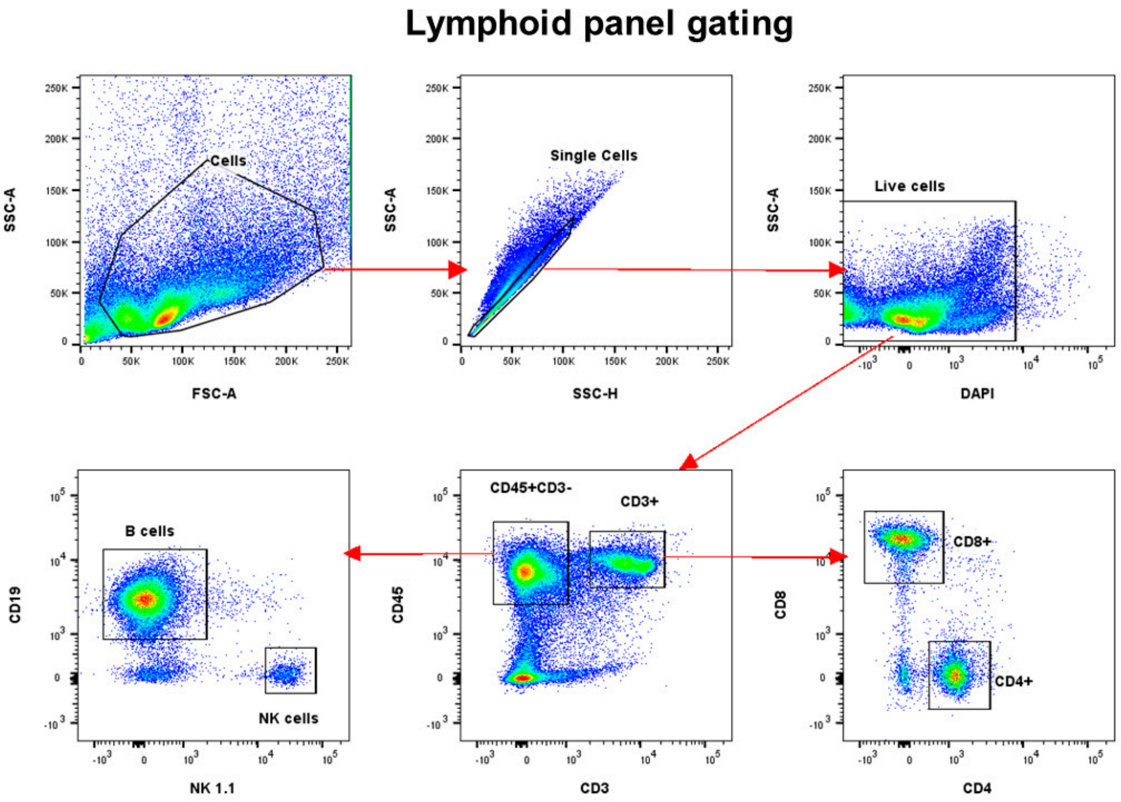

Supplementary Figure S4. Gating strategy for flow cytometry of the lymphoid cells. Gating for lymphoid cell population.

Cytotoxic T cell panel gating

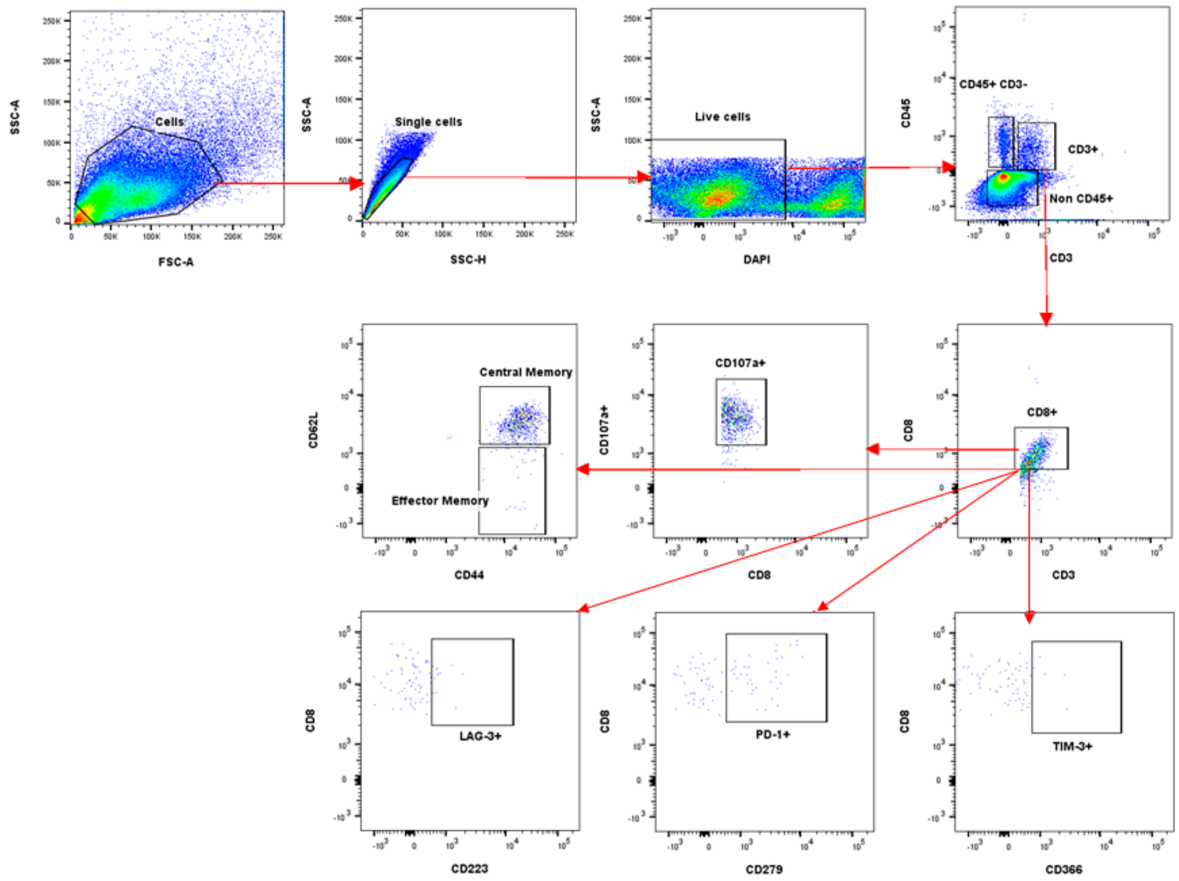

Supplementary Figure S5. Gating strategy for flow cytometry of T cells. Gating for degranulation (CD107a), memory (CD44, CD62L) and exhausted T cells (PD-1, TIM-3, LAG-3).

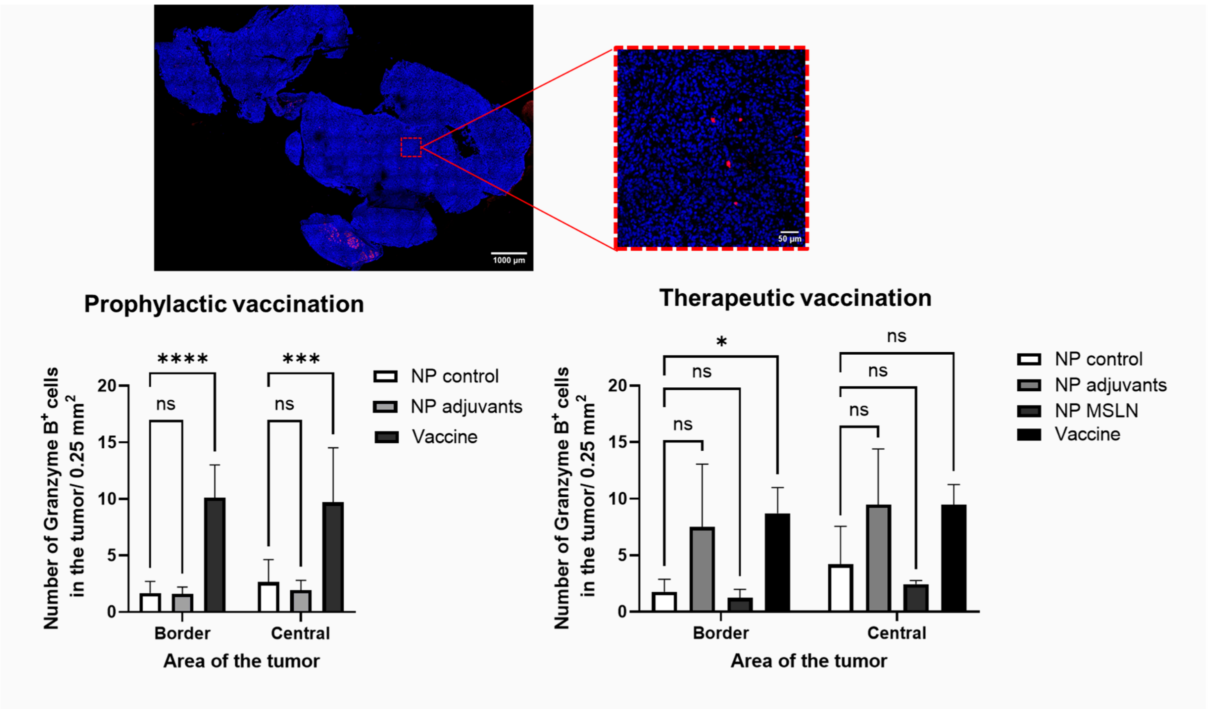

Supplementary Figure S6. Granzyme B+ cells in the tumors. We quantified the presence of Granzyme B^+ cells by immunofluorescence in the border and central areas of the tumors from the prophylactic and therapeutic vaccinations. In the prophylactic vaccination approach, we observed a higher number of Granzyme B^+ cells in both the border and the central areas of the tumor from MSLN nanovaccinated mice, compared to mice that received NP control. For the therapeutic vaccination, mice treated with the MSLN nanovaccine showed an increased number of Granzyme B^+ cells in the border areas of the tumors, in comparison to the NP control group. n= 3-6 per group. Two-way ANOVA, followed by Sidak's multiple comparisons was performed, *$p<0.05$, ****$p<0.0001$, ns = not significant.

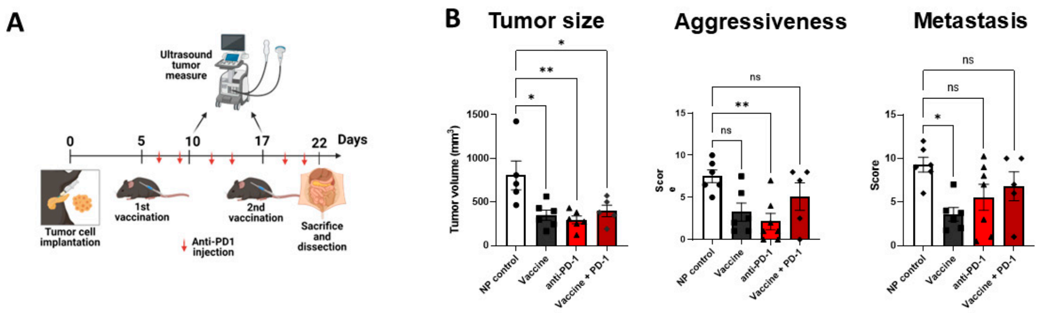

Supplementary Figure S7. Combination treatment with MSLN nanovaccine and anti-PD-1. For this, we orthotopically implanted C57BL/6 mice with KPC cells, then they were divided into four groups according to the treatment: NP control, MSLN nanovaccine, anti-PD-1 antibody, and MSLN nanovaccine + anti-PD1. (A) Mice were treated with nanovaccine after 5 and 17 days of tumor cell implantation. In addition, mice received anti-PD-1 therapy twice per week. Mice were sacrificed on the 22nd day. (B) MSLN nanovaccine reduced tumor size and metastasis score, in comparison to the NP control group. Mice that received only anti-PD-1 therapy also showed a reduction in tumor size and lower aggressiveness score, compared to the control group. The combination therapy of the MSLN nanovaccine and anti-PD-1 resulted in smaller primary tumors than in NP control-treated mice, but similar tumor sizes to the single treatments (nanovaccine or anti-PD-1 alone). In addition, mice treated with the combination had aggressiveness and metastasis scores that were almost as high as those of NP controls. n=5-7 per group. One-way ANOVA, followed by Sidak's multiple comparisons. *$p<0.05$, **$p<0.01$, ns = not significant.
